# Recovery Colleges Characterisation and Testing in England (RECOLLECT): rationale and protocol

**DOI:** 10.1186/s12888-022-04253-y

**Published:** 2022-09-24

**Authors:** Daniel Hayes, Claire Henderson, Ioannis Bakolis, Vanessa Lawrence, Rachel A. Elliott, Amy Ronaldson, Gabrielle Richards, Julie Repper, Peter Bates, John Brewin, Sara Meddings, Gary Winship, Simon Bishop, Richard Emsley, Daniel Elton, Rebecca McNaughton, Rob Whitley, David Smelson, Katy Stepanian, Merly McPhilbin, Danielle Dunnett, Holly Hunter-Brown, Caroline Yeo, Tesnime Jebara, Mike Slade

**Affiliations:** 1 Health Service and Population Research Department, David Goldberg Centre, King’s College London Institute of Psychiatry, Psychology and Neuroscience, De Crespigny Park, London, SE5 8AF UK; 2 South London and Maudsley NHS Foundation Trust, Denmark Hill, London, SE5 8AZ UK; 3 Department of Biostatistics and Health Informatics, King’s College London Institute of Psychiatry, Psychology and Neuroscience, De Crespigny Park, London, SE5 8AF UK; 4 Manchester Centre for Health Economics, Division of Population Health, Health Services Research and Primary Care, School of Health Sciences, Faculty of Biology, Medicine and Health, The University of Manchester, Oxford Road, Manchester, M13 9PL UK; 5 ImROC-Implementing Recovery for Organisational Change, Nottinghamshire Healthcare NHS Foundation Trust, Duncan Macmillan House, Porchester Road, Mapperley, Nottingham, NG3 6AA UK; 6 Peter Bates Associates, 96 Burlington Road, Nottingham, NG5 2GS UK; 7 Nottingham Healthcare NHS Foundation Trust, Duncan MacMillan House, Porchester Rd, Mapperley, Nottingham, NG3 6AA UK; 8 University of Nottingham School of Education, Nottingham, NG8 1BB UK; 9 Nottingham University Business School, Nottingham, NG8 1BB UK; 10 RECOLLECT Lived Experience Advisory Panel, Nottingham, UK; 11 Department of Psychiatry, Douglas Research Centre, McGill University, 6875 LaSalle Boulevard, Montreal, Quebec H4H 1R3 Canada; 12 Department of Psychiatry, University of Massachusetts Medical School, 55 Lake Avenue, North Worcester, Massachusetts, 01655 USA; 13 School of Health Sciences, Institute of Mental Health, University of Nottingham, Nottingham, NG7 2TU UK; 14 Nord University, Postboks 474, 7801 Namsos, Norway

**Keywords:** Recovery College, Effectiveness, Cost-effectiveness, Outcomes, Mechanisms, Mental health, Recovery, Adult learning, Co-production, Fidelity

## Abstract

**Background:**

Recovery Colleges are a relatively recent initiative within mental health services. The first opened in 2009 in London and since then numbers have grown. They are based on principles of personal recovery in mental health, co-production between people with lived experience of mental health problems and professionals, and adult learning. Student eligibility criteria vary, but all serve people who use mental health services, with empirical evidence of benefit. Previously we developed a Recovery College fidelity measure and a preliminary change model identifying the mechanisms of action and outcomes for this group, which we refer to as service user students. The Recovery Colleges Characterisation and Testing (RECOLLECT) study is a five-year (2020–2025) programme of research in England. The aim of RECOLLECT is to determine Recovery Colleges’ effectiveness and cost-effectiveness, and identify organisational influences on fidelity and improvements in mental health outcomes.

**Methods:**

RECOLLECT comprises i) a national survey of Recovery Colleges, ii) a prospective cohort study to establish the relationship between fidelity, mechanisms of action and psychosocial outcomes, iii) a prospective cohort study to investigate effectiveness and cost-effectiveness, iv) a retrospective cohort study to determine the relationship between Recovery College use and outcomes and mental health service use, and v) organisational case studies to establish the contextual and organisational factors influencing fidelity and outcomes. The programme has been developed with input from individuals who have lived experience of mental health problems. A Lived Experience Advisory Panel will provide input into all stages of the research.

**Discussion:**

RECOLLECT will provide the first rigorous evidence on the effectiveness and cost effectiveness of Recovery Colleges in England, to inform their prioritising, commissioning, and running. The validated RECOLLECT multilevel change model will confirm the active components of Recovery Colleges. The fidelity measure and evidence about the fidelity-outcome relationship will provide an empirically-based approach to develop Recovery Colleges, to maximise benefits for students. Findings will be disseminated through the study website (researchintorecovery.com/recollect) and via national and international Recovery College networks to maximise impact, and will shape policy on how Recovery Colleges can help those with mental health problems lead empowered, meaningful and fulfilling lives.

## Background

Personal recovery has been described as the subjective process of taking control of one’s life and one’s mental health, having hope for the future and taking personal responsibility for one’s own recovery [1–4]. The aim of personal recovery is to live as well as possible despite the impact of any continuing symptoms. A shift in mental health services towards supporting personal recovery is recommended internationally [5] and is now central to healthcare policy in many countries [6–11].

Recovery Colleges are a relatively recent initiative within the mental health service system. The first one was piloted in 2009 and officially opened in 2010 in South West London [12, 13]. Drawing on pioneering work in the United States and ideas such as the expert patient programme in the UK [14], they support people with mental health problems, their carers, and mental health staff, via adult-education-based approaches rather than clinical or therapeutic models [12]. Key principles are that they are collaborative, strengths-based, person-centred, inclusive and community-focused [13, 15, 16]. Recovery Colleges have opened in at least 22 different countries in Europe, Asia, Africa, North America and Oceania [13, 17]. An international community of practice has also developed which has met in person twice in recent years [18].

In the UK, a Recovery College model has been developed with a particular focus on co-production and educational co-learning [12, 13]. Some organisations use this model but under other names, such as discovery centre, empowerment college or recovery academy. Educational co-learning involves learning from each other’s life experience and researching what works best, underpinned by a values-oriented approach and clear learning objectives rather than therapeutic approaches [18]. Individuals with lived experience co-produce all aspects of the Recovery College including curriculum development, quality assurance and course delivery, alongside trainers with professional or topic-specific expertise [19]. Students who attend Recovery Colleges can select courses to attend, sometimes with informal supporters. Courses offered vary in content and length, covering both mental health related and other topics, such as: understanding different mental health problems and treatment options; self-management skills; developing skills and confidence to get the most out of services; capacity building and developing the peer workforce; physical health oriented classes (e.g. yoga or swimming); rebuilding life, including courses on seeking, obtaining and staying in employment and helping people to provide support for family members and friends who experience mental health problems.

Best estimates suggest that annually around 36,000 students were enrolled at Recovery Colleges in the UK by 2017 [15] when there were around 80 Recovery Colleges in England. They span a wide range of settings, including primary, secondary, inpatient, and forensic care, and across the voluntary, statutory and private sectors.

### Recovery colleges: What is the evidence?

A recent review identified 31 peer reviewed publications on the impact of Recovery Colleges, from England (*n* = 24), Australia (*n* = 5), Canada (*n* = 1) and Italy (*n* = 1) [20]. Thirteen studies used qualitative methods, ten used quantitative and eight used mixed methods. The review identified service user student and Recovery College staff benefits and initial economic evidence.

#### Student and service provider level benefits

Reported benefits for service user students include increased confidence and self-esteem [16, 19], reduced self-stigma and sense of identity [21], hope, new skills and knowledge [22], improved social networks [23], healthier lifestyle adoption [24], quality of life [25, 26], wellbeing [26], and achieving goals, particularly regarding education [22].

Mental health staff attending Recovery Colleges either as trainers or as students have reported increased knowledge and skills, along with attitudinal changes [27]. The experience of co-production is described as stimulating a renewed motivation for their work generally, and particularly for working more collaboratively with service users. For some, it resulted in decisions to change the language they used and in how they shared information with service users [28].

While promising, these results cover a small proportion of Recovery Colleges and many studies are from single sites using uncontrolled cross-sectional or pre-post designs [29]. These methodological limitations make strong conclusions hard to draw [20]. Given the heterogeneity and rapid expansion of Recovery Colleges nationally and internationally, there is a need for multi-site longitudinal evaluations to better understand their effectiveness.

#### Service use and economic benefits

There have been no formal economic evaluations of Recovery Colleges, and costing studies have focused on the impact on service use, rather than wider potential economic effects such as employment. Uncontrolled studies suggest that attendance is associated with reduced hospital and community service use [30, 31]. One such study found that Recovery College use was followed by a reduction in bed days, involuntary admissions, and community contacts over 18 months [30]. Reductions were greater for those students who had completed a course than those who had only registered. Authors estimated cost savings of UK£1,200 per registered student and UK£1,760 for students who completed a course. A further study suggested similar benefits [31]. A recent Australian costing study examined differences in costs of mental health community hours, mental health inpatient admissions and Emergency Department presentations that occurred before and after attending the Recovery College, in combination with top-down costs of the programme [32]. They reported overall net savings as A$269 per service user student per year in the 12 months post-enrolment, but also remarked on the challenges of comparisons across different funding arrangements for individual colleges.

### The RECOLLECT programme

Recovery Colleges Characterisation and Testing (RECOLLECT) is a programme of research investigating Recovery Colleges in England. It is funded by the National Health Service (NHS) National Institute of Health Research (NIHR), through the Programme Grants for Applied Research scheme, and runs from 2020 to 2025. Students other than those who are or have used mental health services are outside the remit of the RECOLLECT programme.

#### Theory informing the RECOLLECT Programme

The RECOLLECT Programme is based on foundational work completed in an earlier funded study in England in 2017. Three empirically-based knowledge products were developed as a first step to more rigorously study Recovery Colleges.

First, the RECOLLECT Fidelity Measure [33] is a quantitative standardised assessment of Recovery College fidelity, i.e. the extent to which the Recovery College is organised according to key components. These components were identified through a systematized search and narrative literature review (13 publications) and semi-structured interviews with Recovery College managers across England (*n* = 10). An initial checklist was commented on by four expert groups (*n* = 77 in total): members of ImROC, the national transformation programme supporting recovery and wellbeing practice (*n* = 7); the Recovery College International Community of Practice (*n* = 54) comprising international experts in developing or evaluating Recovery Colleges; a Lived Experience Advisory Panel (LEAP) comprising mental health service user Recovery College students and nonstudents, and family members (*n* = 9); and the RECOLLECT International Advisory Board (IAB) (*n* = 7). It was then refined through semi-structured interviews with Recovery College students, trainers, and managers (*n* = 44) in three sites. The RECOLLECT Fidelity Measure was adapted from the checklist and evaluated with Recovery College managers (*n* = 39/75, a 52% response rate), clinicians providing psychoeducational courses (*n* = 11), and adult education lecturers (*n* = 10). The 12-item measure comprises seven non-modifiable components (Valuing equality; Learning; Tailored to the student; Co-production of the Recovery College; Social connectedness; Community focus; and Commitment to recovery) and five modifiable components (Available to all; Location; Distinctiveness of course content; Strengths-based; and Progressive). The measure meets scaling assumptions and demonstrates adequate internal consistency (0.72), test–retest reliability (0.60), content validity and discriminant validity, specifically differentiating between Recovery Colleges, adult education colleges and clinician-run psychoeducational groups. Rasch analysis found that Coproduction of the Recovery College and Learning were the most likely components to be endorsed, indicating that use of coproduction and adult learning approaches are foundational aspects of fidelity. The RECOLLECT Fidelity Measure is available at www.researchintorecovery.com/recollect.

**Fig. 1:**
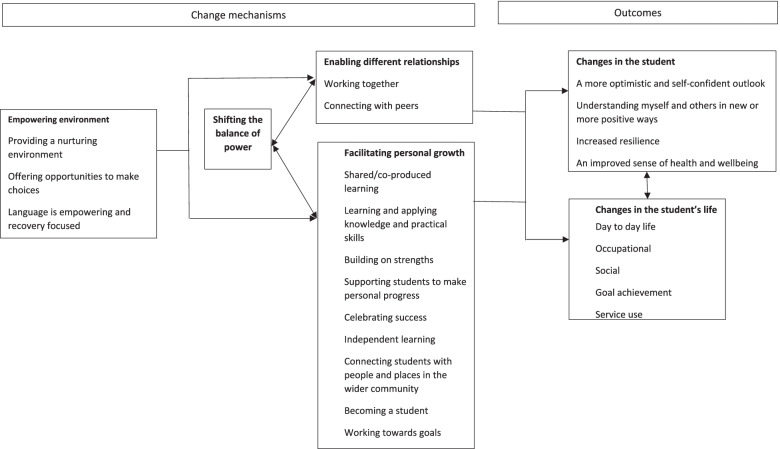
RECOLLECT change model for service user students

Second, a RECOLLECT change model describes the impact of Recovery Colleges for service user students in terms of mechanisms of action leading to outcomes [16]. The model was developed initially through collaborative data analysis [34] by research team members and lived experience co-researchers integrating findings from 44 documents identified in a systematised review. It was refined and validated through qualitative interviews with service user students (*n* = 12), local community partners (*n* = 7), peer trainers (*n* = 4), clinician trainers (*n* = 3), recovery college managers (*n* = 3) and mental health commissioners, clinicians and managers (*n* = 4). Four mechanisms of action were identified: empowering environment; shifting the balance of power; enabling different relationships; and facilitating personal growth. Outcomes for service user students comprised intrapersonal change such as increased optimism and self-confidence, and changes in the student’s life such as in their social network or employment status. The content of each component and the relationship between components of the resulting change model are shown in Fig. 1.

Finally, the impact of Recovery Colleges at other levels – mental health staff, organisational level and wider society – was investigated [27]. Using the same methods as the RECOLLECT change model for the service user student level, a stratified theory was developed identifying candidate mechanisms of action and outcomes for Recovery Colleges at staff, services and societal levels. At the staff level, experiencing different relationships may change attitudes and associated professional practice. Identified outcomes for staff included: experiencing and valuing co-production; changed perceptions of service users; and increased passion and job motivation and a change in language used. At the organisational level, Recovery Colleges often involve a degree of autonomy from their parent organisation, for example the provision of education rather than treatment. This allows development of an alternative culture giving experiential learning opportunities to staff around co-production and the role of a peer workforce. Beyond the host organisation, at the level of the wider society, Recovery Colleges partner with community-based organisations. This gives other members of the public opportunities for learning alongside people with mental health problems and enables such organisations to work with people they might not have otherwise, creating opportunities to improve mental health literacy and reduce mental health related stigma [35].

**Fig.2:**
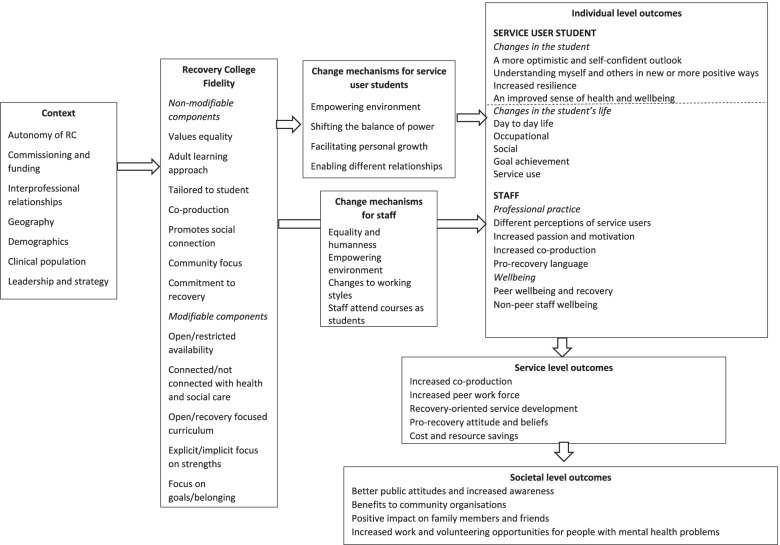
RECOLLECT multi-level change model

The change model for the service user student level and the stratified theory for impact at other levels were integrated to form a preliminary RECOLLECT multilevel change model, shown in Fig. 2.

## Aims and objectives

The aims of the RECOLLECT Programme are to establish the effectiveness and cost-effectiveness of Recovery Colleges in England, and to identify how Recovery Colleges can be optimised to maximise the benefit for people with mental health problems.

Specific objectives, with the abbreviations used hereafter in brackets, are:

1. To describe Recovery Colleges in England (Characterise)
2. To establish Recovery College costs (Costs)
3. To investigate changes over time in service user student outcomes for an inception cohort across multiple Recovery Colleges (Student outcomes)
4. To investigate the relationships between fidelity, mechanisms of action and outcomes for an inception cohort (Fidelity, mechanisms and outcome)
5. To assess the effectiveness of Recovery Colleges for an inception cohort (Effectiveness)
6. To assess the cost effectiveness of Recovery Colleges for an inception cohort (Cost-effectiveness)
7. To explore the relationship between Recovery College use and routine clinical NHS data (Clinical data) for a retrospective cohort
8. To establish the key contextual and organisational factors influencing fidelity and variation in outcomes (Organisational)
9. To finalise the RECOLLECT multilevel change model (Change model)
10. To use findings to maximise Recovery College effectiveness and coverage (Knowledge mobilisation).

## Methods

### Design

**Fig.3:**
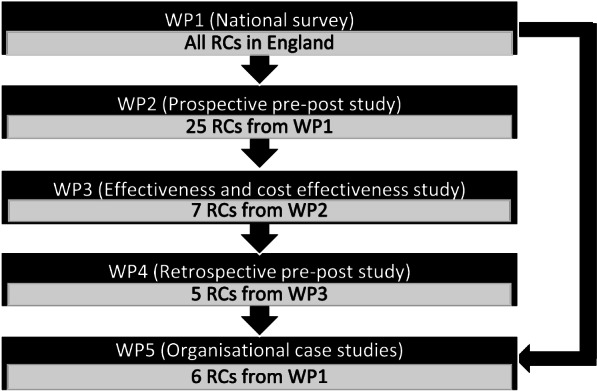
RECOLLECT Work Packages

The RECOLLECT programme comprises six work packages structured to relate to the RECOLLECT multilevel change model. Work Package 1 is a national survey of managers of Recovery Colleges in England, supplemented by qualitative interviews with a subsample of survey respondents. Work Package 2 is a prospective pre-post study of Recovery College fidelity and service user student outcomes and service use. Work Package 3 is a prospective controlled study comparing outcomes and service use among newly registered Recovery College service user students with a matched service user control group. Work Package 4 is a retrospective controlled study comparing routine clinical data for service user students with a matched control group. Work package 5 is a series of organisational case studies. Work Package 6 will mobilise the resulting knowledge from Work Packages 1 to 5. The inter-relationships and objectives addressed in Work Package 1 to 5 are shown in Fig. 3.

### Recovery college sample

The inclusion criteria for Recovery Colleges are:

1. A focus on supporting personal recovery in mental health or substance use;
2. An aspiration to use co-production, defined as individuals with lived experience working with staff or subject experts to design and deliver all aspects of the Recovery College;
3. An aspiration to use adult learning approaches, in which students and trainers collaborate and learn from each other by sharing experiences, knowledge, and skills;
4. Location in England.

These criteria were agreed by a Recovery College stakeholder group, consisting of academics, LEAP members involved with Recovery Colleges, Recovery College Managers, and members of ImROC, which supports recovery college development as part of its partnership work on recovery and wellbeing practice. The rationale for the criteria was based on: the common purpose of Recovery Colleges to support personal recovery, the foundational importance of co-production and an adult learning approach identified through analysis of the fidelity measure data [33] and the geographical scope of the programme. The organisation did not need to be called a ‘Recovery College’ to be included, since some use other names such as discovery centre, empowerment college or recovery academy.

### Project management

The RECOLLECT programme is jointly led by King’s College London and University of Nottingham, and researchers will be employed at both sites. The applicant team comprises 14 members with expertise in all aspects of the programme. The Programme Steering Committee (PSC) comprises six independent members to ensure governance and independent oversight. The International Advisory Board (IAB) will maximise the scientific quality of the programme, fair and impartial evaluation of Recovery Colleges, and contribute to dissemination. The 11 IAB members have multidisciplinary and cross-cultural expertise in all programme aspects. The Lived Experience Advisory Panel (LEAP) comprises 10 individuals who either have lived experience of mental health problems or are carers, some but not all of whom have used Recovery Colleges. To ensure that expertise by experience informs all aspects of the programme, the LEAP will provide input into choice of measures, support design decision-making, inform documentation for participants, interview participants, be involved in interpretation of data, and assist with publicising findings. Lived experience is also represented on the Programme Steering Committee, International Advisory Board and in the research team.

Work packages 1 to 6 are now described.

### Work package 1: National survey and qualitative interviews

Work package 1 will address Objectives 1 (Characterise) and 2 (Costs).

#### Design and rationale

Work Package 1 consisted of a national survey of all Recovery College managers [36] and qualitative interviews with a subsample of managers. In addition to addressing Objectives 1 and 2, results will be used to design the sampling frame for Work Packages 2 and 5, inform the economic evaluations in Work packages 3 and 4, and describe the impact of the COVID-19 pandemic on Recovery Colleges. In relation to this latter aim, the COVID-19 pandemic began after the RECOLLECT Programme was funded. When the programme began in December 2020, the long-term impact of the pandemic on Recovery Colleges was not clear, but the short-term impact was high, as many Recovery Colleges temporarily closed and all moved from primarily or exclusively face-to-face provision to entirely online provision during periods of national lock-down. The design of Work Package 1 was therefore supplemented with semi-structured qualitative interviews with Recovery College managers, to understand the impact of COVID-19 as a contextual influence on Recovery Colleges, including its impact on fidelity.

#### Sample

The survey sample is managers of Recovery Colleges in England, per the above criteria. The semi-structured interviews will be conducted with a sub-group of survey participants. It is anticipated that saturation [37] will be achieved through no more than 40 interviews.

#### Measures

The RECOLLECT Fidelity Measure is a 12-item Recovery College manager-rated assessment [33]. It comprises seven non-modifiable components (Valuing equality; Learning; Tailored to the student; Co-production of the Recovery College; Social connectedness; Community focus; and Commitment to recovery) and five modifiable components (Available to all; Location; Distinctiveness of course content; Strengths-based; and Progressive). Each non-modifiable component is scored on a three-point scale from 0 (low fidelity) to 2. Each modifiable component is rated as either Type 1 or Type 2 (e.g. eligibility restricted to those affiliated with the host organisation, vs available to all), and as each modifiable component is independent, Recovery Colleges can score Type 1 on some components and Type 2 on others. No summary score is produced, since the relationship between fidelity and outcome is not empirically established.

#### Procedures

To maximise coverage, Recovery Colleges were identified using multiple approaches: web searches containing key words related to Recovery Colleges and geographical area, advertising of the survey through social media and Recovery College networks, involvement of study partner organisations such as ImROC, and snowball sampling. All Recovery College managers were then invited to participate by email and provided with an information sheet. After giving informed consent and responding to screening questions to ensure their organisation meets the inclusion criteria, managers of those screening as eligible were asked to complete the online survey.

The survey covered characteristics of the Recovery College structure, courses, students, fidelity, and costs. Attention was paid to capturing both current and pre-pandemic data where relevant, and free-text elaboration of responses was allowed. The survey was implemented online on Qualtrics, with an equivalent Microsoft Excel version as an alternative where participants cannot access Qualtrics due to organisational firewalls. The survey was piloted with a small convenience sample of Recovery College managers and refined.

The semi-structured qualitative interview topic guide covers history, organisational context, COVID-related changes and future plans. Interviews were conducted by RECOLLECT researchers, LEAP members who will receive qualitative interviewer training and supervision, and RECOLLECT research team members with expertise in qualitative methodologies and organisational sociology. Recovery College managers who participate in the national survey were asked to participate in a (maximum) one-hour semi-structured interview either online or by telephone. After the consent process, the interview was conducted, with notes kept by the interviewer on their impressions and key discussion points. Interviews will be audio recorded, transcribed and anonymised.

#### Analysis

Recovery College characteristics were investigated using descriptive analysis exploring RECOLLECT Fidelity Measure scores and numerical characteristics such as student numbers. Hierarchical cluster analysis was used to create a national Recovery College typology. Content analysis of any free text survey responses and collaborative data analysis [34] using framework analysis [38, 39] of interview transcripts will be employed to explore organisational characteristics and pandemic impact. To assess costs, the expenses associated with Recovery Colleges will be identified and quantified, including salaries of those employed by the Recovery College and by other organisations who release employees as sessional trainers, building and IT costs, curriculum development and delivery, and time spent by stakeholders such as service users, informal carers and mental health staff who attend as students [26]. Costs will then be apportioned to specific sectors as appropriate with input and feedback from stakeholders.

### Work package 2: Prospective pre-post study exploring Recovery College impact on student mental health outcomes

Work package 2 will address Objectives 3 (Student outcomes), 4 (Fidelity, mechanisms and outcomes) and 9 (change model).

#### Design and rationale

Work Package 2 will consist of a prospective pre-post study at 25 Recovery Colleges selected from the survey sample to provide the full range of Fidelity Measure item scores. The change model and input from LEAP informed the development of items to assess the mechanisms of action and the choice of outcome measures, i.e. the primary outcome of quality of life and secondary outcome measures covering intrapersonal changes and changes to the students’ life including changes in service use. In addition, we will use the generic health status measure, EQ-5D-5L [40] to allow comparison of the samples in Work Package 2 and Work Package 3, and hence assess generalisability of the intervention group in Work Package 3.

#### Sample

To address Objective 3 we have conservatively assumed a small but clinically significant within-person effect size of 0.2 [41]. With standard deviation (SD) of 5 based on the mean difference of total score of the primary outcome measure, the Manchester Short Assessment (MANSA) [42] between baseline and follow up at 12 months with 95% power and 5% significance, assuming an intra-class correlation coefficient (ICC) of 0.02 [43, 44], an individual autocorrelation of MANSA score over time of 0.5 [45] and within-cluster standard deviation (SD) of 5, we require a sample size of 500. With an estimate of 20% attrition rate by 12 months, we will recruit 625 participants, for an analysable sample of 500. We will recruit 625 newly registering students (25 per college) who currently use secondary mental health services. Exclusion criteria will be insufficient knowledge of English to complete the outcome measures.

#### Measures

The primary outcome is quality of life as measured by the MANSA [46]. This comprises 12 items rated using a seven-point Likert scale, with higher scores indicating higher quality of life. The MANSA has been shown to have good psychometric properties, including internal consistency and validity [42, 47]. Secondary outcomes comprise: empowerment in relation to mental health service use, measured by the Mental Health Confidence Scale [48]; personal recovery measured by Brief INSPIRE-O [49]; social inclusion measured by the Social Inclusion Scale/Measure (Social inclusion) [50]; resilience measured by the Brief Resilience Scale [51]; hope as measured by the Herth Hope Index [52]; Social network size using the Lubben Social Network Scale 6 [53]; and mental wellbeing, using the Warwick-Edinburgh Mental Wellbeing Scale (Short version) [54].

Student service use will be measured using a Service Use Questionnaire designed for the project and will capture both NHS and non-NHS service use. The SUQ covers contacts with health and social care, use of leisure facilities, employment, income and out of pocket expenses. We will include the number and types of RC courses attended. To provide a societal perspective, it will be tailored to cover work status and absence, sources of income, domestic activities, informal care, medication use, out-of-pocket expenses, and consumption of non-medical resources. Where consent is given by students, mental health clinical records will be accessed to supplement findings.

The EQ-5D-5L is a self-rated measure of health-related quality of life [40]. The scale measures quality of life on a 5-component scale including mobility, self-care, usual activities, pain/discomfort, and anxiety/depression. Psychometrically, it is less prone to ceiling effects than the EQ-5D-3L across a range of diseases [55, 56]. The National Institute for Health and Care Excellence (NICE) requires use of a 5L to 3L crosswalk algorithm for their reference-case to estimate QALYs [57].

#### Procedures

All new students will be made aware of the research by Recovery College staff as they register. Those who express interest in participating will be contacted by a member of the research team who will provide the participant information sheet either remotely or in person. Participation will be optional, and students can choose to register with the college and not take part in the RECOLLECT Study. Eligible students who provide informed consent will then complete baseline measures, sociodemographic data and provide follow-up contact information for re-administration of all measures at 4, 8, and 12 months. Clinical and service use data will be collected from electronic medical records given participant consent for access; this consent will be optional for those consenting to participate in measure completion.

#### Analysis

To address Objective 3 (Student outcomes), we will compare primary outcome (total MANSA score) at baseline, 4, 8, and 12 months follow-up adjusted for baseline total MANSA score and accounting for hierarchical clustering of students at the level of Recovery College. The model will include the MANSA score as the outcome variable, baseline MANSA score and socio-demographic characteristics as explanatory variables at each time point. Specifically, we will fit a three-level random intercept linear regression model, considering observations from baseline, 4, 8 and 12 months at level 1, individuals at level 2 and Recovery Colleges at level 3. Secondary outcomes will be assessed similarly to the primary outcomes, using generalized linear mixed models (logistic, linear and Poisson depending on the distribution of our outcome variable).

The relationship between Fidelity Measure scores at the Recovery College level with primary and secondary outcomes will also be assessed using generalised linear mixed models. Where significant associations emerge, theoretical mechanisms of action will be added to models to examine potential mediating effects.

If indicated, we will refine the RECOLLECT multilevel change model by removing outcomes where there is no evidence of change at any time point.

### Work package 3: Prospective controlled study exploring the effectiveness and cost effectiveness of Recovery Colleges

Work package 3 will address Objectives 5 (Effectiveness) and 6 (Cost-effectiveness).

#### Design and rationale

Work package 3 will employ a prospective controlled study design and include seven of the Recovery Colleges from Work Package 2, with which it will run in parallel. A non-randomised design will be employed due to the impossibility of individual level randomisation; most mental health service users have access to a Recovery College, even if not available in their Trust. The seven Recovery Colleges were selected for being linked to an NHS Trust which has access to a Clinical Record Interactive Search (CRIS) system, as this will be used to identify the control group.

#### Sample

Intervention group eligibility will be as for Work Package 2; eligible controls will not be attending a Recovery College at baseline, or have previously attended a Recovery College. Work Package 3 will require approximately 48 students at each of the seven Recovery Colleges to form an intervention group of 330 participants; and 660 participants in total with the control group. This sample size is needed for 90% power to detect an effect size of 0.2 on the MANSA [46] using a two-sided test for the difference between two independent means and assuming α = 0.05. To meet this recruitment target, students at the seven Recovery College sites for Work Package 3 will be oversampled (i.e. 48 instead of 25 per site) relative to the others in Work Package 2.

#### Settings

The seven participating Recovery Colleges linked to an NHS Trust with access to a CRIS system cover areas within five of the nine regions of England defined by the Office for National Statistics: South West, South East, East and North West England and London.

#### Measures

Work Package 3 will use the same primary and secondary outcome measures as for Work Package 2.

#### Procedures

**Fig. 4:**
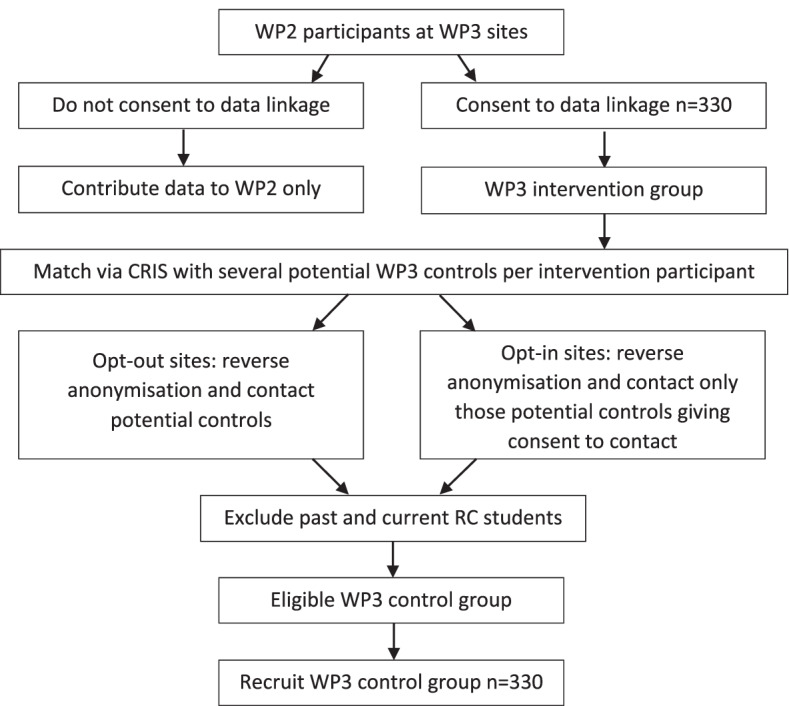
Relationship between Work Packages 2 and 3 at Work Package 3 site

Work Package 3 will run alongside Work Package 2. At the seven Recovery College sites involved in Work Package 3, participants will be asked if they would like to take part in Work Package 3 by consenting to data linkage using CRIS. Those that consent will comprise the intervention group for Work Package 3. This is outlined in Fig. 4 below. To identify the control group, propensity score matching [58] will be employed using the CRIS system at each site. This is a statistical matching technique that attempts to create a comparable control group for the Recovery College intervention group by accounting for the covariates, such as demographics and clinical history, that predict outcomes. Reverse anonymisation will be undertaken by CRIS staff and potential control participants will be contacted by NHS Trust staff working with CRIS staff to find out if participants are interested in hearing more about the study. Those that are will be provided with information sheets and will need to give informed consent to take part. The intervention group will complete those measures used in Work Package 2 and listed above at the same time points of baseline, 4, 8 and 12 months. The control group will complete the primary outcome measure and a restricted set of those measures used in Work Package 2 and the Work Package 3 intervention group, to reduce burden. Participants in Work Packages 2 and 3 will be compensated for their time completing assessments.

#### Analysis

We will compare the total MANSA score for service users who attended a Recovery College (intervention group) with those who did not (control group) at each follow up, controlling for baseline scores and hierarchical clustering of patients in Recovery Colleges. Specifically, we will fit a three-level random intercept linear regression model, considering observations from baseline, 4, 8 and 12 months at level 1, individuals at level 2 and Recovery Colleges at level 3. The model will include the MANSA scores at 4, 8 and 12 months as the outcome variables with intervention group, baseline MANSA score and sociodemographic characteristics, including the matching factors. A time x intervention interaction will be included to allow the effect to differ at each time point. Those secondary outcomes collected in both the intervention and control groups will be assessed similarly to the primary outcomes, using generalized linear mixed model (linear, logistic and Poisson depending on the distribution of the outcome.

Incremental economic analyses will be conducted from both a healthcare and a societal perspective with analyses conducted at 4, 8 and 12-month follow-up, controlling for baseline. Uncertainty will be addressed by generating cost-effectiveness planes from bootstrapped resamples. Cost effectiveness acceptability curves will examine the probability that the intervention is cost-effective for different decision-maker willingness-to-pay thresholds, as well as generating net benefit estimates. Sensitivity analysis will investigate key factors including the impact of costs of different Recovery College delivery models and the use of EQ-5D-5L/EQ-5D-3L cross-walk methods to generate utility [57]. The benefits of Recovery Colleges are likely to continue after the 12-month primary endpoint. Therefore, we will also carry out an economic evaluation informed by modelling to estimate longer-term benefits and NHS/Personal and Social Service and wider costs, using current design quality standards [59]. This requires an expert consensus panel to support model design, made up of Lived Experience Advisory Panel, Recovery College staff and the applicants, and representatives of Trusts and other affiliated organisations.

### Work package 4: Retrospective controlled study of the effectiveness of Recovery Colleges

Work Package addresses Objective 7 (clinical data).

#### Design and rationale

Work Package 4 will employ a retrospective controlled study using routine clinical data and measures, with an 18 month follow up period. In comparison to Work Package 3, this study will benefit from a larger sample size and absence of selection bias in the intervention group, to produce results that will be more generalisable across NHS sites.

#### Measures

The Health of the Nation Outcomes Scale (HoNOS) [60] is a 12-item measure completed by clinical staff at all NHS mental health Trusts which covers a patient’s health and social functioning. Each item is scored on a five-point Likert scale. It has moderately high construct validity, fair to moderate test–retest reliability and good predictive validity [61].

DIALOG is an 11-item measure exploring health status and quality of life used routinely in many NHS mental health trusts, and based on the MANSA [62]. There are eight items on quality of life and three items on treatment, each scored on a seven-point Likert scale. Internal consistency is 0.71 for the eight quality of life measures and 0.57 for the three treatment items [62].

The Clinical Outcomes in Routine Evaluation – Outcome Measure (CORE-OM) [63] is a 34 item self-report measure covering subjective wellbeing, symptoms, function and risk used in many NHS mental health trusts. Each item is scored on a four-point Likert-scale. Internal consistency and test–retest reliability are good (0.75–0.95) [64].

#### Procedures

**Fig. 5:**
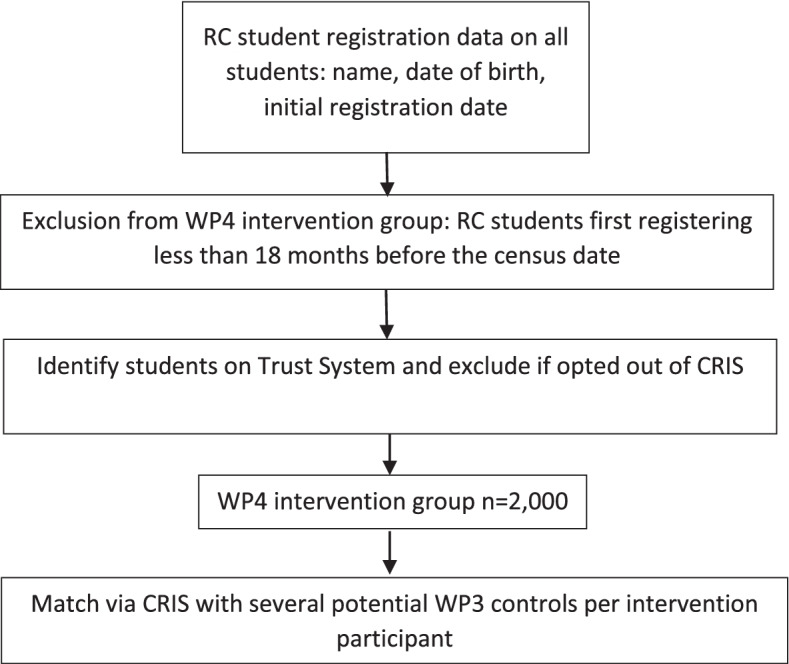
Process for participant identification in Work Package 4

Five of the seven Recovery College sites used in Work Package 3 will be employed for this study (see Fig. 5), based on availability of student data and affiliation with an NHS Trust which has a CRIS. At each site service user student and CRIS data will be linked.

The intervention group will comprise all service user students who first registered at the Recovery College over 18 months prior to the selected audit date (the date of the data extraction from the Recovery College). Based on student numbers and the proportion who are service users we estimate 400 per site (total *n* = 2,000).

Individual service users in the control group will be matched with the students using a similar propensity scoring approach to that described in Work Package 3, so that as far as possible the only difference between groups will be Recovery College attendance. For the intervention group Recovery College registration will be used as the baseline date, and the last HoNOS and other available outcome scores recorded before Recovery College registration will be used as the baseline data. Eligible controls will also have a HoNOS score at the equivalent timepoint. The follow up HoNOS and other outcome scores to be used will be those entered as an update at the closest time point to the 18 month follow up point. For both groups, data on service use will be extracted for the 18 months after the baseline, including number of psychiatric inpatient days; number of contacts with staff in community mental health teams and specialist services such as psychology and outpatient services; and medications including doses prescribed.

#### Analysis

We will fit a two-level random intercept linear regression model, considering observations from individuals at level 1 and Recovery Colleges at level 2. We will compare HONOS scores of intervention group individuals at 18 months with 18-month HoNOS for matched control individuals adjusting for baseline HONOS and sociodemographic characteristics. The model will include 18 months HoNOS score as the outcome variable, and the baseline HoNOS score and sociodemographic characteristics as explanatory variables.

The relationship between Recovery College use, and mental health service use will be examined using both resource consumption parameters (categorical data) and derived individual-level costs (continuous data). Individual level costs will be calculated by combining service use data with appropriate unit costs, using the methods outlined in Work Package 1. Costs will be compared for the intervention group compared with matched controls using a bootstrapped regression model, adjusting for HoNOS and other outcome measures as available.

### Work package 5: Organisational case studies of Recovery Colleges

Work Package 5 addresses Objectives 8 (Organisational) and 9 (Change model).

#### Design and rationale

Work Package 5 will consist of organisational case studies. It will include six Recovery Colleges from Work Package 2, to be purposefully sampled to capture variation in Fidelity Measure score, length of time in operation, size, and funding arrangements. Within the constraints of maximum variation sampling and the need to manage burden on each Recovery College, we will prioritise Recovery Colleges which were also sites for Work Packages 3 and 4; this will enhance finalisation of the multilevel change model given the evidence from these sites on effectiveness and cost effectiveness.

#### Procedures

Qualitative data collection using in-depth interviews and focus groups will be undertaken with stakeholders: Recovery College Trust managers; peer and non-peer trainers; and commissioners, and past and present students, including but not limited to service user students. For interviews of professionals, snowball sampling will be used, starting with the Recovery College manager. Students will be recruited via Recovery College staff, as well as posters, emails and flyers. Topic guides tailored to the participant will explore: how contextual and organisational factors influence fidelity; the mechanisms proposed in the RECOLLECT change model leading to outcomes and how fidelity influences these mechanisms. Content will include: 1) manager and other staff views on what influences fidelity items; 2) student experiences of attending, using probes based on Fidelity Measure items; 3) observations by those in contact with Trust services (service users, carers and staff) of any changes they have observed in the Trust since the Recovery College opened which they think may be influenced by the presence of the college, and 4) stakeholder views about the impact of the Recovery College on the local community.

#### Analysis

Collaborative data analysis involving LEAP members again using Framework analysis [38, 39] will be employed to facilitate analysis within and between groups of participants, using NVivo [65]. Context will be explored by learning from experiences of managing organisational interfaces and commissioning arrangements. Cross-case comparison will elaborate the key contextual and organisational factors influencing the delivery of service and explaining variation in access, fidelity and outcome. Analysis will focus on the relationships posited in the multilevel change model.

### Work package 6: Knowledge mobilisation

#### Design and rationale

RECOLLECT will produce: 1) An evidence-based understanding of what a Recovery College is, captured by the RECOLLECT Fidelity Measure; 2) A contextualised understanding of how Recovery Colleges work, expressed through the RECOLLECT multilevel change model; and 3) the first rigorous evidence about the effectiveness and cost effectiveness of Recovery Colleges.

The following key stakeholders will be informed of the findings: Recovery College students, mental health service staff, policy makers, commissioners, researchers, research participants, and the general public. Dissemination will include tailored messaging through various networks (professional, service user and carer, research networks including in low- and middle-income countries, ImROC, the Recovery College Network), international Recovery College community of practice online including through social media and the study website (researchintorecovery.com/recollect), and through a knowledge mobilisation event at the end of the programme. Outputs will include papers in high impact academic and practitioner journals, posters, conferences, reports and briefings.

## Discussion

The comprehensive and rigorous mixed-method approach taken in the RECOLLECT programme has the potential to fill the gaps in the literature identified in the introduction. This has a number of potential benefits. First, national and international use of the RECOLLECT Fidelity and multilevel change model will guide the implementation of Recovery Colleges to optimise the quality of provision. Second, application of the RECOLLECT multilevel change model has the potential to increase Recovery College efficiency, both by reducing waste due to ineffective components and by facilitating mechanisms through which Recovery Colleges benefit service users. These mechanisms include actions by Recovery Colleges, for example promoting access to other community organisations, and by host organisations, such as increasing the peer workforce, ensuring staff are given time to attend, or increasing co-production. Potential benefits of these actions include practice changes on the part of staff who attend the college as students or trainers, leading to greater use of shared decision making and public stigma reduction though contact with the peer workforce and greater mental health literacy. Third, evidence of effectiveness and cost effectiveness would justify increased Recovery College access for those who wish to attend and ensure the sustainability and expansion of Recovery Colleges in England and elsewhere. Fourth, evidence of effectiveness and cost effectiveness would stimulate exploration of their applicability to other long term health conditions. Finally, our results will lead to further research based on the multilevel change model. We will update the study website (researchintorecovery.com/recollect) with progress, and further collaborations around the RECOLLECT programme are welcomed in order to establish a more rigorous evidence base for Recovery Colleges. 

## Data Availability

The quantitative datasets in Work Packages 1, 2 and 3 will be available from the corresponding author on reasonable request. The quantitative dataset from Work Package 4 and the qualitative datasets from Work Packages 1 and 5 will not be publicly available. For Work Package 4 this is due to the CRIS access arrangements. For qualitative data this is due to the difficulty of anonymisation.

## Abbreviations

CRIS: Clinical Record Interactive Search
CSRI: Client Service of Receipt
CORE-OM: The Clinical Outcomes in Routine Evaluation – Outcome Measure
HoNOS: The Health of the Nation Outcomes Scale
IAB: International Advisory Board
LEAP: Lived Experience Advisory Panel
MANSA: The Manchester Short Assessment
NHS: National Health Service
NICE: National Institute for Health and Care Excellence
NIHR: National Institute for Health Research
RECOLLECT: Recovery Colleges Characterisation and Testing
SD: Standard Deviation
WP: Work Package
UK: United Kingdom
